# Genes controlling root development in rice

**DOI:** 10.1186/s12284-014-0030-5

**Published:** 2014-11-28

**Authors:** Chung D Mai, Nhung TP Phung, Huong TM To, Mathieu Gonin, Giang T Hoang, Khanh L Nguyen, Vinh N Do, Brigitte Courtois, Pascal Gantet

**Affiliations:** Agricultural Genetic Institute, LMI RICE, Hanoi, Vietnam; University of Science and Technology of Hanoi, LMI RICE, Hanoi, Vietnam; IRD, UMR DIADE, LMI RICE, Hanoi, Vietnam; CIRAD, UMR AGAP, Montpellier, France; Université Montpellier 2, UMR DIADE, Montpellier, France

**Keywords:** Rice, Crown root, Lateral root, Branching, QTL mapping, Association mapping, Gene cloning, Direct genetics, Reverse genetics

## Abstract

In this review, we report on the recent developments made using both genetics and functional genomics approaches in the discovery of genes controlling root development in rice. QTL detection in classical biparental mapping populations initially enabled the identification of a very large number of large chromosomal segments carrying root genes. Two segments with large effects have been positionally cloned, allowing the identification of two major genes. One of these genes conferred a tolerance to low phosphate content in soil, while the other conferred a tolerance to drought by controlling root gravitropism, resulting in root system expansion deep in the soil. Findings based on the higher-resolution QTL detection offered by the development of association mapping are discussed. In parallel with genetics approaches, efforts have been made to screen mutant libraries for lines presenting alterations in root development, allowing for the identification of several genes that control different steps of root development, such as crown root and lateral root initiation and emergence, meristem patterning, and the control of root growth. Some of these genes are closely phylogenetically related to *Arabidopsis* genes involved in the control of lateral root initiation. This close relationship stresses the conservation among plant species of an auxin responsive core gene regulatory network involved in the control of post-embryonic root initiation. In addition, we report on several genetic regulatory pathways that have been described only in rice. The complementarities and the expected convergence of the direct and reverse genetic approaches used to decipher the genetic determinants of root development in rice are discussed in regards to the high diversity characterizing this species and to the adaptations of rice root system architecture to different edaphic environments.

**Electronic supplementary material:**

The online version of this article (doi:10.1186/s12284-014-0030-5) contains supplementary material, which is available to authorized users.

## Introduction

Roots are essential organs for exploring and exploiting soil resources, such as water and mineral nutrients. Different root architecture ideotypes that are adapted to different soil mineral nutrient balances or water statuses have been proposed. It is generally acknowledged that a deeper, thicker and more branched root system with a high root to shoot ratio can enhance the tolerance of rice to water deficits (Fukai and Cooper, [1995]; Gowda et al. [2011]). Roots develop primarily underground and are inaccessible to direct observation. This limitation partly explains why root architecture is not a widespread breeding objective. Because of this lack of past investment, there is room for improvement of rice root systems. This task is particularly important in a context of climate change, which will likely increase the occurrence of unfavorable conditions (Den Herder et al. [2010]). An improved root system can be obtained more easily using indirect selection methods, such as marker-assisted selection. It is therefore necessary to identify the main genetic determinants governing root development. Root development is a complex process that involves constitutive and adaptive mechanisms and regulatory correlations with the shoot part of the plant (Puig et al., [2012]). In this review, we focus on genetic determinants of root development in rice that were identified using complementary direct and reverse genetic approaches, such as QTL detection, mutant analysis and transcriptomics. The involvement of these genes in plant adaptation to stress conditions is discussed when such information is available and relevant.

## Review

### Main characteristics of the rice root system

The rice root system is composed of a seminal root and postembryonic shoot borne-roots termed crown roots (Rebouillat et al. [2009]; Coudert et al. [2010]). These two types of roots can branch and form long lateral roots or short lateral roots. Crown roots differentiate in the stem from a radial ground meristem that has common characteristics with the root pericycle (Coudert et al. [2013b]; Itoh et al. [2005]). Lateral roots differentiate from the root pericycle and, in part, from the endoderm (Rebouillat et al. [2009]; Orman-Ligeza et al. [2013]). The radial structure of the rice roots comprises the following tissues, from the center to periphery: the stele, including phloem and xylem vessels and the pericycle; the endoderm; the cortex, whose cells can undergo apoptosis to constitute the aerenchyma; the sclerenchyma; the exodermis; and the epidermis (Rebouillat et al. [2009]). This radial structure reflects the capacity of rice roots to grow in aerobic as well as anaerobic conditions. Notably, the aerenchyma enables gas exchange with the shoot when the plant is growing under anaerobic conditions. Rice was domesticated approximately 10 000 years ago (Sweeney and McCouch [2007]) and has since adapted to a large variety of ecosystems (irrigated, rainfed lowland, upland, flood-prone, and mangrove) and production systems (from traditional low-input systems to intensive high-input systems). More than 100 000 rice genotypes are available in the Genebank of the International Rice Research Institute (IRRI), Philippines. The phenotypic evaluation of a small sample of this diversity showed that the tested genotypes exhibited a great deal of root architecture variation (O’Toole and Bland [1987]; Lafitte et al. [2001]). The remaining unexplored extensive variability constitutes a very significant opportunity for the identification of new genes involved in root development.

### Detection of QTLs for root development in rice

**Figure 1:**
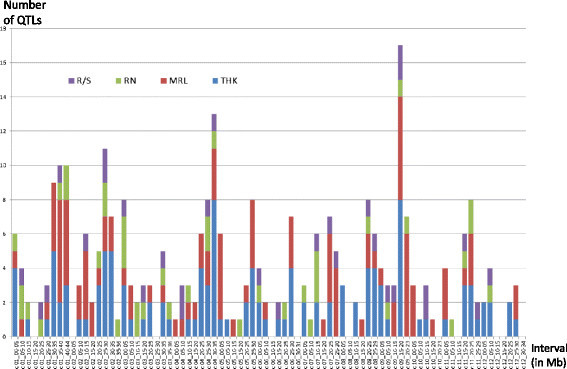
**Number of root related QTLs detected in rice per chromosome interval.** QTL for root to shoot ratio (R/S, violet), root number (RN, green) maximum root length (MRL, red), and root thickness (THK, blue). The figure was built from data reported in Courtois et al. ([2009]).

To identify genes controlling root system architecture, the direct genetic approach has historically preceded the reverse genetic approach. The direct genetic approach was first based on QTL detection in mapping populations. The first study on root architecture in rice was undertaken in 1995 (Champoux et al. [1995]), and a large number of studies followed. These studies were reviewed by Kamoshita et al. ([2008]) for those concerning the CT9993/IR622266 population and [Khowaja et al. (2009)] for those concerning Bala/Azucena. Courtois et al. ([2009]) analyzed the conditions of detection of 675 QTLs in 24 studies conducted on 12 different populations. Simple phenotyping systems, generally based on hydroponic culture conditions, pots or soil columns, characterized this period of work. In the majority of the cases (368 among the 675 QTLs), these phenotyping conditions were favorable in order to estimate the crop’s genetic potential; only a few studies specifically analyzed response QTLs in conditions of water stress or root penetration constraints (147 and 160 QTLs, respectively). The measured traits were those that were the most easily observable, such as maximum root length, root thickness, root number, mass of roots at different depths, or root to shoot ratio. The preferred mapping populations were recombinant inbred lines (RILs) or doubled haploid populations because the fixed nature of these lines enabled replicate measurements. To maximize the phenotypic differences between parents and simplify the identification of polymorphisms, most of the populations (8 populations representing 560 QTLs) were derived from indica x japonica crosses. The mapping population sizes were generally relatively small (between 100 and 150 accessions), enabling the identification of several QTLs with large effects but offering little resolution. Although large QTL x environment interactions were recorded (Kamoshita et al. [2002]; MacMillan et al. [2006]), some chromosomal regions appeared to be detected regularly in various populations and/or environment, as shown by meta QTL analyses (Courtois et al. [2009]; [Khowaja et al. 2009]). For the traits for which the highest number of QTL were detected, QTL clusters were found on chromosomes 1 (30–40 Mb), 2 (25–35 Mb), 3 (0–5 Mb), 4 (30–35 Mb), and 9 (15–20 Mb), as shown in Figure 1. More recently, the progress on high-throughput root phenotyping and 3D image reconstruction has led to a renewed interest in QTL detection in mapping populations, focusing on original traits (e.g., the root network bushiness or its convex hull volume), in addition to more classical traits, such as specific root length or specific root area (Topp et al. [2013]). This last study using the Bala/Azucena population revealed QTLs that had already been detected for simpler traits and identified new QTLs as well.

The main limitation with QTL detection in these mapping populations is the large size of the confidence interval. Even meta-analyses, on average, did not enable a decrease in the meta-QTL confidence intervals to less than half the original size of the segment (Courtois et al. [2009]). Genome-wide association studies (GWAS) in natural populations showing fast linkage disequilibrium decay have recently appeared as powerful tools to improve the resolution in localizing QTLs compared to mapping populations (Zhu et al. [2008]). GWAS requires a very high marker density, which is permitted by the simultaneous development of next-generation sequencing technology. The first results of GWAS analyzing root traits in rice have recently been published (Clark et al. [2013]; Courtois et al. [2013]). Because of the strong bipolar structure of *O. sativa*, which induces high risk of false-positive associations, the analyses were conducted on panels composed of accessions belonging to a single varietal group. This type of panel gives access to new varietal group-specific QTLs that cannot be detected by classical mapping in indica x japonica populations. However, even if the resolution on QTL position is clearly improved in GWAS compared with mapping populations, the number of possible candidate genes is still large and requires complementary evidence to determine the functional gene(s).

### Positional cloning of genes underlying root QTLs

The results of QTL mapping were pursued through the development of NILs introgressed with QTLs in different backgrounds and QTL fine mapping with the aim of positional cloning (Shen et al. [2001]; Steele et al. [2013]; Suji et al. [2012]; Steele et al. [2006]). The most interesting QTLs for which the importance of the phenotypic effect was confirmed were positionally cloned. The first of these was *PHOSPHORUS UPTAKE 1* (*PUP1*), a QTL contributing to phosphorus (P) uptake in low P content soils. The gene underlying the QTL, later termed *PHOSPHORUS-STARVATION TOLERANCE 1* (*PSTOL1*), was cloned and appeared to encode a receptor-like cytoplasmic kinase (Gamuyao et al. [2012]). The gene does not exist in Nipponbare. When overexpressed, the gene significantly increases the plant P content and enhances grain yield by more than 60% under P-deficient conditions. The gene acts by enhancing early root growth, conferring a globally larger root system to the plant, with enhanced root length and root surface area, contributing to an increased uptake of P, and of other nutrients (N and K). The gene is expressed in the zone of crown root initiation and crown root meristems in stem bases. This localization suggests that the gene is involved in the regulation of crown root formation and elongation.

Another cloned gene related to a root development QTL is *DEEPER ROOTING 1* (*DRO1*), which controls root growth angle and enhances deep rooting (Uga et al. [2013]). *DRO1* encodes an unknown protein that is associated with plasmalemma. *DRO1* expression is regulated by auxin via AUXIN RESPONSE FACTOR (ARF) transcription factors. DRO1 modulates root gravitropic response, likely via a modulation of epidermal cell elongation that enables roots to orientate their growth relative to the pull of gravity. The expression of *DRO1* in the IR64 background increases the angle between roots and the horizontal axis, inducing deeper rooting. Compared to IR64, the near isogenic lines carrying *DRO1* showed less deleterious effects, such as leaf wilting or delayed flowering, under moderate to severe drought stresses and better yield under stress, with no yield penalty under no stress conditions.

The cloning of these two QTLs that directly or indirectly affect root architecture exemplifies the difficulties that can occur in QTL cloning and can partly explain why the number of cloned root QTLs is still low. The first difficulty, which is intrinsic to the cloning process whatever the trait, is the lack of precision in positioning QTLs in mapping populations or, to a much lesser extent, in association panels. Therefore, lengthy operations are needed to reduce the size of the intervals to the gene level. A second difficulty in gene cloning is due to the importance of structural differences between varietal groups (Schatz et al. [2014]) and, within varietal groups, between varieties, as is illustrated by the absence of genes such as *PSTOL1* in the reference cultivar Nipponbare. A final reason is that many genes do not have an assigned function. For example, the *DRO1* product has no similarity to known proteins (Uga et al. [2013]). For these reasons, it is essential to complement these genetic studies that exploit the rice diversity using functional analysis approaches.

**Figure 2:**
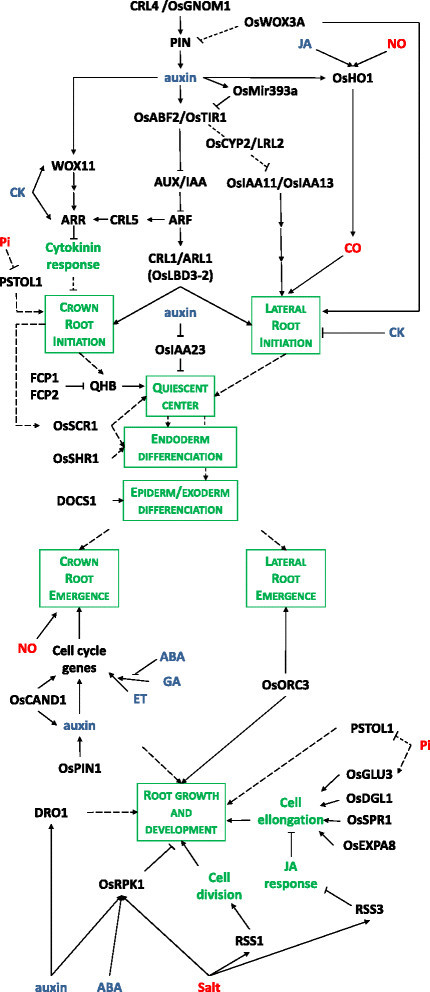
**Gene networks controlling root development in rice** ***.*** Arrows represent the positive regulatory action of one element of the network on another one. A line ending with a trait represents the negative regulatory action of one element of the network on another one. Dotted line represent hypothetical link between two elements. Text color code: genes, black; hormones, blue; signals, red; biological processes, green.

The sequencing and annotation of the rice genome (Itoh et al. [2007]; Yu et al. [2002]) as well as the creation of mutant collections (Hirochika et al. [2004]; Krishnan et al. [2009]; Wei et al. [2013]; Lorieux et al. [2012]; Guiderdoni and Gantet [2012]) and their screening for altered root phenotypes allowed the identification of major genes regulating the initiation, emergence or development of rice roots (Rebouillat et al. [2009]; Coudert et al. [2010]; Orman-Ligeza et al. [2013]). The gene networks involved in rice root development discussed below are schematized in Figure 2.

### A conserved core gene regulatory network regulates post-embryonic root initiation in response to auxin

The *crown root less 4* (*crl4*) mutant is impaired in *OsGNOM1*, which encodes a membrane-associated guanine-nucleotide exchange factor for the ADP-ribosylation factor G protein (Kitomi et al. [2008]; Liu et al. [2009]). This latter factor is a homolog of GNOM1, which regulates the intracellular traffic of the PINFORMED1 (PIN1) auxin efflux carrier protein in *Arabidopsis*. In turn, PIN1 is necessary to concentrate auxin in lateral root funder cells (Kitomi et al. [2008]; Liu et al. [2009]; Steinmann et al. [1999]). *Crl4* and *Osgnom1* mutants are characterized by an absence of crown roots and a reduced number of lateral roots. In these mutants, the expression of several *OsPIN* genes is altered. In the *crl4* mutant, auxin transport is impaired, causing auxin distribution to be modified in the stem bases (Kitomi et al. [2008]). This result suggests that local auxin transport is involved in the initiation of crown and lateral roots in rice. WUSCHEL-related homeobox 3A (OsWOX3A) is encoded by *NARROW LEAF2* (*NAL2*) and *NAL3*, a pair of duplicated genes. The *nal2*/*nal3* double mutant shows a complex phenotype, with altered development of different parts of the plant and, notably, a strong reduction of the density of lateral roots. This latter effect is associated with an increase in root hair number and length (Cho et al. [2013]). In *nal2*/*nal3* mutants, the expression of different *OsPIN* genes is modified. It is hypothesized that this modification results in a preferential allocation of auxin to the epidermis, where root hair formation is promoted. This occurs to the detriment of the pericycle, where lateral roots initiate (Yoo et al. [2013]). *OsTIR1* and *OsAFB2*, the rice orthologs of the *Arabidopsis* auxin receptors TRANSPORT INHIBITOR RESPONSE 1(TIR1) and AUXIN SIGNALING F-BOX2 (AFB2), are negatively regulated by *OsMir393a* and *OsMir393b* (Bian et al. [2012]; Xia et al. [2012])*.* Plants overexpressing *OsMir393* exhibit a pleiotropic altered auxin signaling phenotype, including a reduced number of crown roots. *OsMir393a* is expressed in the initiation sites of crown and lateral roots, suggesting that *OsMir393a* is involved in the regulation of post-embryonic root formation via a negative regulation of the rice auxin receptors. Interestingly, the TIR1 and AFB2 auxin receptors are localized to the nucleus, where they can interact with OsIAA1, an AUXIN/INDOLE ACETIC ACID (AUX/IAA) regulatory protein (Bian et al. [2012]; Xia et al. [2012]). AUX/IAA proteins interact with and inhibit the auxin response factors (ARF) that function as transcription factors. In *Arabidopsis*, when the TIR1 receptor is complexed with auxin, it can interact with AUX/IAA proteins. This binding results in the recruitment of a SKP1-CULLIN-FBOX (SCF) ubiquitin ligase, which ubiquitinates the AUX/IAA protein, targeting it for degradation. These interactions require other chaperone and co-chaperone proteins (Guilfoyle and Hagen [2007]; Vanneste and Friml [2009]; Mockaitis and Estelle [2008]; Lavenus et al. [2013]). In rice, a mutant screen for a loss of auxin sensitivity resulted in the characterization of two mutants, *Oscyclophilin2* (*Oscyp2*) and *lateral rootless2* (*lrl2*), which are characterized by an absence of lateral roots and are caused by mutations in the same gene (Kang et al. [2013]; Zheng et al. [2013]). In addition, *Oscyp2* exhibits a pleiotropic auxin-insensitive phenotype that correlates with a reduced degradation of OsIAA11 in response to auxin (Kang et al. [2013]). OsCYP2/LRL2 is a chaperone protein that can interact with the co-chaperone SUPPRESSOR OF G2 ALLELE OF SKP1 (OsSGT1). It has been proposed that this complex is involved in the degradation of OsIAA11 by SCF^TIR^ in response to auxin during the early step of lateral root initiation (Kang et al. [2013]). OsIAA11 or OsIAA13 gain of function mutations, in which amino acids involved in degradation of the protein in response to auxin are modified, exhibit a lateral rootless phenotype (Kitomi et al. [2012]; Zhu et al. [2012]). OsIAA11 and OsIAA13 are required for lateral root initiation. The *crown root less 1* (Inukai et al. [2005]) and *adventitious less 1* (Liu et al. [2005]) mutants are both impaired in the *OsLBD3-2* gene, which encodes a LATERAL ORGAN BOUNDARY DOMAIN (LBD) transcription factor belonging to a specific clade regrouping LBD proteins involved in the control of post-embryonic root initiation in different species (Coudert et al. [2013a]). *Crl1* and *arl1* mutants are characterized by a total absence of crown root initiation and a reduced number of lateral roots. The expression of *CRL1* is induced by auxin. This induction is lost in plants overexpressing a modified OsIAA3 protein that cannot be degraded after auxin treatment, showing that the induction of the expression of *CRL1* by auxin is dependent on the degradation of AUX/IAA proteins. In addition, the *CRL1* promoter contains an auxin response sequence (*ARE*) that interacts *in vitro* with the OsARF16 transcription factor (Inukai et al. [2005]). Taken together, these data show that the initiation of crown and lateral roots in rice involves a core auxin regulated pathway that includes TIR1/AFB2 auxin receptors, AUX/IAA regulatory proteins, as well as specific ARF and LBD transcription factors. This gene regulatory network that controls post-embryonic root development is well conserved in different dicot and monocot plant species (Peret et al. [2009]; Lavenus et al. [2013]; Hochholdinger and Tuberosa [2009]). In rice, some elements of this regulatory network are common for the initiation of crown and lateral roots, whereas others are specific to the initiation of only one root type. For this reason, rice constitutes a promising model to investigate how this auxin-dependent gene regulatory network has evolved to control post-embryonic root development in the context of both stems and roots (Coudert et al. [2013b]).

### Post-embryonic root initiation in rice involves genes that mediate other hormone signals

The *crown root less5* (*crl5*) mutant exhibits a reduced number of crown roots due to a reduction in crown root initiation (Kitomi et al. [2011]). *CRL5* encodes an APETALA/ETHYLENE RESPONSE FACTOR (AP2/ERF) transcription factor. Its expression is induced by auxin via an ARF transcription factor. Cytokinins negatively regulate the initiation of crown and lateral roots in rice (Rani Debi et al. [2005]; Kitomi et al. [2011]), but plants that constitutively express CRL5 can initiate crown roots normally. CRL5 activates the expression of two type-A *RESPONSE REGULATORs* (*ARRs*) that are repressors of cytokinin signaling (To et al. [2004]). Similarly, the WUSCHEL-related Homeobox transcription factor WOX11 controls the expression of different *ARRs* and regulates both crown root initiation and development (Zhao et al. [2009]). *WOX11* expression is induced by auxin and cytokinin. This result shows that *CRL5* and *WOX11* are involved in the crossway of the antagonistic auxin and cytokinin pathways in the regulation of crown-root initiation.

The *HEME OXYGENASE 1* (*HOX1*) gene controls the formation of lateral roots via the production of carbon monoxide. *HOX1* is regulated by auxin and stress related signals, such as jasmonate and nitric oxide (Chen et al. [2012]; Hsu et al. [2012]), which suggests that this pathway can contribute to the modulation of root architecture in response to stress.

### Genes involved in root meristem formation

After the initiation step, cells divide and become organized to shape a root meristem. In root meristems, the quiescent center (QC), which is composed of a small number of mitotically inactive cells, is indispensable for the maintenance of the undifferentiated status of the surrounding stem cells from which root tissues are produced (van den Berg et al. [1997]). The maintenance by auxin of the QC in rice is mediated by OsIAA23 (Jun et al. [2011]). The *QUIESCENT CENTER HOMEOBOX* (*QHB*) gene is an ortholog to the *WUSCHEL*-related *WOX5*, a QC-specific gene in *Arabidopsis* that contributes to QC and root stem cell maintenance (Kamiya et al. [2003b]; Breuninger et al. [2008]; Ditengou et al. [2008]). *QHB* is expressed early during root formation in the QC, and its expression is dependent on *CRL1/ARL1* during crown root formation (Inukai et al. [2005]; Ni et al. [2014]; Kamiya et al. [2003b]; Liu et al. [2005]). The CLAVATA3 (CLV3)/ENDOSPERM SURROUNDING REGION (ESR)-related (CLE) FON2-LIKE CLE PROTEIN1 (FCP1) and FCP2 proteins inhibit the expression of *QHB* in the QC (Chu et al. [2013]; Suzaki et al. [2008]). This relationship suggests that a CLAVATA/WOX signaling module regulates the activity of the root apical meristem in rice. Such a module has already been described in *Arabidopsis* for the regulation of the shoot apical meristem activity. In this context, the transduction of the CLV3 peptide signal is ensured by LEUCINE RICH REPEAT RECEPTOR-LIKE KINASE (LRR RLK) CLV1 (Muller et al. [2006]). In rice lines overexpressing *OsRPK1*, which encodes an LRR RLK, the expression levels of different *OsPIN* genes are down regulated, polar auxin transport is perturbed and the length of the seminal root is reduced, as is the number of crown roots and the density of lateral roots (Zou et al. [2014]). Nevertheless, it is undetermined at which step of root development (i.e., initiation, meristem maintenance or other development processes) this gene is involved. Interestingly *OsRPK1* is regulated by auxin, abscisic acid and salt stress. Its role in stress-adaptive root morphogenesis should be further studied (Zou et al. [2014]).

In *Arabidopsis*, SCARECROW (SCR), a transcription factor from the GRAS family, also contributes to the specification of the QC (Sabatini et al. [2003]). In rice, *OsSCR1* is expressed in the QC and may also be involved in QC determination (Ni et al. [2014]). The expression of *OsSCR1* is down-regulated in stem bases of the *arl1* mutant (Liu et al. [2005]). Together with SHORTROOT (SHR, another GRAS transcription factor), SCARECROW controls the division of the endoderm/cortex initial cells and the differentiation of endoderm in *Arabidopsis* (Di Laurenzio et al. [1996]; Cui et al. [2007]). In rice, this mechanism is likely conserved given that *OsSCR1* is also expressed in endoderm/cortex initial cells and in early differentiated endodermal cells (Ni et al., [2014]). In addition, OsSCR1 can interact with OsSHR1, which is expressed in the stele but may likely move to endodermal initial cells (Cui et al. [2007]; Ni et al. [2014]; Kamiya et al. [2003a]). Rice roots are characterized by an exodermis as well as sclerenchyma cell layers just inside the epidermis (Rebouillat et al. [2009]; Coudert et al. [2010]). An aluminum-sensitive mutant, *c68*, has been identified (Huang et al. [2009]). In this mutant, the differentiation of the epidermis and exodermis is perturbed. The corresponding gene, *DEFECTIVE IN OUTERCELL LAYER SPECIFICATION 1* (*DOCS1*), encodes an LRR RLK protein (Huang et al. [2012]). This result illustrates that a better knowledge of genes involved in root meristem functioning and root radial tissue patterning should help identify new sources of resistance and improve the adaptation of rice to soil containing toxic elements.

### Genes involved in post-embryonic root emergence

When formed, the root meristem must grow out through the stem (for crown roots) or the root (for lateral roots) tissues. Crown root emergence is stimulated by submergence. This stimulation is mediated by ethylene, which induces the expression of cell cycle regulatory genes in crown root meristems and promotes, in synergy with gibberellic acid, the death of epidermal cells at the site of root emergence (Lorbiecke and Sauter [1999]; Mergemann and Sauter [2000]; Steffens et al. [2006]). Nitric oxide also promotes crown root emergence, whereas abscisic acid inhibits emergence (Steffens et al. [2006]; Xiong et al. [2009]). A functional auxin polarized transport, largely mediated by OsPIN1, is also required for crown root emergence and development (Xu et al. [2005]). In the *Oscand1* mutant, crown root meristems are properly initiated and formed but do not emerge (Wang et al. [2010]). In *Arabidopsis*, *OsCAND1* encodes a CULLIN-ASSOCIATED AND NEDDYLATION-DISSOCIATED 1 (CAND1) SCF^TIR1^ ubiquitin ligase involved in the degradation of AUX/IAA in response to auxin (Cheng et al. [2004]). In the *Oscand1* mutant, auxin distribution is altered in crown root meristems, as is the expression of cell cycle regulatory genes. In *origin recognition complex subunit3* (*orc3*) knock-down plants, the emergence of lateral roots is blocked due to a perturbation of cell cycle activity in newly formed lateral root meristems (Chen et al. [2013]). *OsORC3* is also expressed in primary root and crown root primordia, likely playing a more general role in root system development. Root emergence and root initiation are essential processes that determine the number of developed roots of the plant. In this regard, it is also essential to identify the major genetic determinants governing this process to engineer more branched root systems.

### Genes involved in root development

Different root development mutants permitted the identification of genes involved in different biological functions. Some of these genes alter root development by affecting root cell wall constitution. *OsDGL1* encodes DOLICHYL DIPHOSPHOOLIGOSACCHARIDE-PROTEIN GLYCOSYLTRANSFERASE 48 kDa subunit precursor necessary for the proper structuration of the polysaccharide matrix of root cell walls. *Osdgl1* mutant plants exhibit a short root phenotype due to a defect in root cell elongation and division (Qin et al. [2013]). Another gene affecting root cell wall constitution was isolated using short root mutant screening. This gene, *OsGLU3*, encodes a putative MEMBRANE-BOUND ENDO- 1,4-*B*-GLUCANASE (Zhang et al. [2012b]). In *Osglu3* mutants, the crystalline cellulose content is reduced in the root cell wall and root cell elongation is inhibited. It has been suggested that *OsGLU3* can contribute to the modulation of root growth in response to phosphate starvation (Zhang et al. [2012a]). Expansins are also key actors of cell elongation. *OsEXPANSIN8* (*OsEXPA8*) is specifically expressed in root tips (Shin et al. [2005]). Plants overexpressing *OsEXPA8* exhibit better global growth. This improvement is notable in their root system, where root elongation and branching are strongly stimulated (Ma et al. [2013]). This phenotype results in a general increase in cell wall elongation capacity. The *Oryza sativa short postembryonic roots 1* (*Osspr1*) mutant also exhibits a short root phenotype that is due to defective root cell elongation and is characterized by a perturbation in iron homeostasis (Jia et al. [2011]). *OsSPR1* encodes a mitochondrial protein with an Armadillo-like repeat domain, but its precise molecular function is uncharacterized. In the *rice salt sensitive3* (*rss3*) mutant, the elongation of root cells is strongly inhibited under salt stress conditions, resulting in a short root phenotype (Toda et al. [2013]). RSS3 is a nuclear factor that can interact with key molecular components of the jasmonic acid transduction pathway, a stress hormone known to inhibit root growth. In roots, RSS3 likely contributes to repress the jasmonic acid response in salt stress conditions to maintain root growth. Similarly, the *rss1* mutant exhibits strongly reduced root and shoot growth in salt stress conditions (Ogawa et al. [2011]). *RSS1* plays a role in maintaining cell division in meristematic zones under salt stress conditions, likely by interfering with the cytokinin response pathway. The targeted overexpression of NO APICAL MERISTEM, ATAF1-2, CUP-SHAPED COTYLEDON (NAC) transcription factors OsNAC5, OsNAC9 and OsNAC10 in roots enhances the water stress tolerance of plants (Jeong et al. [2010]; Redillas et al. [2012]; Jeong et al. [2013]). The overexpression of these factors correlates with an augmentation in root diameter that may facilitate soil penetration by roots and water transport. All of these genes involved in root growth are promising targets in the creation of deeper root systems that can help plants to avoid water deficits and, as illustrated above in different examples, can modulate root growth in response to different edaphic conditions.

### Transcriptomic studies will facilitate root gene discovery

The first comprehensive transcriptome study of the rice root system was published in 2012 (Takehisa et al. [2012]). In this study, eight developmental stages along the longitudinal axis of a crown root were investigated as well as different radial tissues: epidermis, exodermis and sclerenchyma; cortex; and endodermis, pericycle and stele. This analysis allowed the identification of genes specifically expressed in root caps, in the initiation or developmental zone of lateral roots, and those involved in water and nutrient transport. Data are available in the RiceXPro data base (Sato et al. [2013]). Data comparing genes expressed in roots of different rice varieties have begun being generated (Chen et al. [2014]). Some authors investigated the genes that were miss-regulated relative to wild type plants in rice root-related mutants such as *Osiaa13*, *c68* or *crl1* (Kitomi et al. [2012]; Huang et al. [2012]; Coudert et al. [2011]). In the latter case, the authors performed a second screen based on auxin induction of *CRL1* expression. The aim of this screen was to identify CRL1-dependent auxin regulated genes. One of the identified genes, *FLATTENED SHOOT MERISTEM* (*FSM*) encodes a component of the CHROMATIN ASSEMBLY FACTOR1 that contributes to crown root formation (Abe et al. [2008]). It is clear that comparison of the increasing number of rice root-specific transcriptome data sets obtained using different experimental approaches will allow the identification of new important genes involved in root development in rice.

## Conclusions

In recent years, our knowledge of the genetic control of root development in rice has progressed considerably. Genetic approaches based on the investigation of rice genotypic and phenotypic diversity have allowed the identification of major genes involved in the development of different types of root system architectures, such as *DRO1* and *PSTOL1*, with functions related to drought tolerance or adaptation to soil with low phosphate content, respectively (Gamuyao et al. [2012]; Uga et al. [2013]). The identification of *PSTOL1,* which was only possible using a traditional variety, stresses that the effort to explore the diversity of *O. sativa* that has adapted to a large range of ecosystems must continue. Functional genomics approaches have begun to provide a better understanding of the mechanisms involved in root branching, elongation and radial tissue differentiation. Branching is the fundamental process by which the root system can increase its root number. Together with elongation, branching allows the root system to explore and exploit a larger portion of soil. Radial tissue patterning in rice is fundamental for the adaptation of root growth in anoxic conditions and for the control of the diffusion of toxic elements, such as salt or metals, into roots. The genes governing root meristem activity also constitute promising targets for adapting root growth to different soil stress conditions. Only a better knowledge of the mechanisms that regulate the expression of such genes will allow the design of new breeding strategies that target the root system to improve rice resistance to soil related stresses. The transcriptome analysis of root systems in plants grown in different conditions will help to achieve this objective and will provide a large catalogue of root-specific genes that are expressed at different developmental stages and in response to different stresses.

These approaches (QTL detection, gene functional characterization, global expression studies) need to be more closely linked to accelerate the discovery of new genetic determinants, which will result in the generation of varieties with improved root architecture. We hope that this review helps to link the data obtained by these complementary approaches.

The results of genetic and genomic approaches can converge with the identification of alleles of interest at genes with known function and the introgression of these alleles into elite lines. An example of such a strategy is given by the work conducted on *SUB1*, a gene controlling submergence tolerance, in various Asian countries (Bailey-Serres et al. [2010]). In less than five years, the allele of interest was introgressed into major elite varieties. There is no doubt that the work conducted on root QTLs will lead to the same efforts (Steele et al. [2013]). *DRO1* and *PSTOL1* have begun to be used in several rice improvement-breeding programs. In addition, a targeted diversity analysis of the genes governing different aspects of root growth and development also needs to be conducted. The identification of the allelic variants of genes that are associated with different root system phenotypes should rapidly provide breeders with the necessary tools to finely modulate root architecture in order to address specific needs. However, the very large GxE interactions expected between roots and soil due to variations in physical, chemical and microorganismal soil composition implies that the observed effect of QTL/gene presence in field grown plants might not be as strong as expected. Therefore, there will be significant opportunities for agronomists and farmers to use their skill to optimize the expression of the root potential of the selected varieties under various conditions.

## Authors’ original submitted files for images

Below are the links to the authors’ original submitted files for images. Authors’ original file for figure 1 Authors’ original file for figure 2

## Abbreviations

ABA: Abscissic acid
AFB2: Auxin signaling F-Box2
ARF: Auxin response factor
ARL: Adventitious root less
ARR: Type-A response regulator
AUX/IAA: Auxin/Indole-3-Acetic acid
CAND1: Cullin-Associated and Neddylation-Dissociated 1
CK: Cytokinin
CO: Carbon monoxide
CRL: Crown root less
CYP2: Oscyclophilin2
DRO1: Deeper rooting 1
DGL1: Dolichyl Diphosphooligosaccharide-Protein Glycosyltransfer Glycosyltransferase
DOCS1: Defective in outercell layer specification 1
ET: Ethylene
EXPA8: Expansin8
FCP: Clavata3 (CLV3)/Endosperm Surrounding Region (ESR)-related (CLE) Fon2-Like CLE PROTEIN
GA: Gibberelic acid, GLU3, Membrane-Bound Endo- 1,4-*B*-Glucanase
GNOM1: Membrane-Associated Guanine-Nucleotide Exchange Factor of the ADP-Ribosylation Factor G Protein (ARF-GEF)
HO1: Heme oxygenase 1
JA: Jasmonic acid
LLR2: Lateral root less 2
NO: Nitrite oxide
ORC3: Origin recognition complex Subunit3
Pi: Inorganic phosphate
PIN: Pinformed auxin efflux carrier proteins
RPK1: Receptor protein kinase 1
PSTOL1: Phosphorus-Starvation tolerance 1
QHB: Quiescent center homeobox
RSS: Rice salt sensitive
SCR: Scarecrow
SHR: Shortroot
SPR1: Short postembryonic roots
TIR1: Transport inhibitor response 1
WOX: Wushel-related homeobox
