# Supplementary figures and images for: Genes controlling root development in rice
Source: Rice (N Y). 2014 Nov 28;7:30. doi: 10.1186/s12284-014-0030-5 (PMC4884052; doi:10.1186/s12284-014-0030-5)

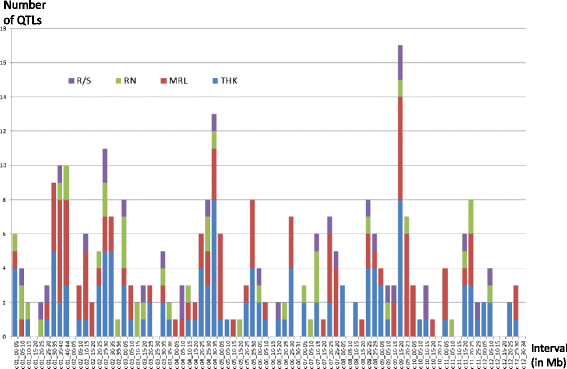

Supplement: Supplementary file 1 — Authors’ original file for figure 1 [file 12284_2014_30_MOESM1_ESM.gif]

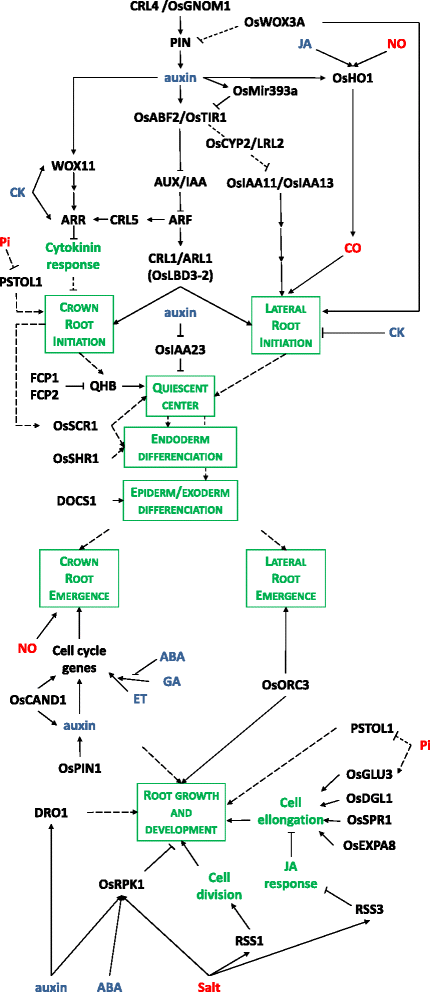

Supplement: Supplementary file 2 — Authors’ original file for figure 2 [file 12284_2014_30_MOESM2_ESM.gif]
